# Mechanical Properties of Solder-Jointed Copper Rods with Electrodeposited Sn-Zn Alloy Films

**DOI:** 10.3390/ma13061330

**Published:** 2020-03-14

**Authors:** Tatsuya Tsurusaki, Takeshi Ohgai

**Affiliations:** 1 Graduate School of Engineering, Nagasaki University, 1-14 Bunkyo-machi, Nagasaki 852-8521, Japan; bb52118641@ms.nagasaki-u.ac.jp; 2 Faculty of Engineering, Nagasaki University, 1-14 Bunkyo-machi, Nagasaki 852-8521, Japan

**Keywords:** Pb-free soldering, Sn-Zn alloy, electrodeposition, citric acid complex, enforced solid solution, thermal annealing, solder-joint, intermetallic compound, tensile strength, stress–strain

## Abstract

Enforced solid solution type Sn-Zn alloy films were electrochemically synthesized on Cu substrate from an aqueous solution containing citric acid complexes. The electrodeposition behavior of Sn-Zn alloys was classified to a normal co-deposition type, in which electrochemically nobler Sn deposits preferentially compared to Zn. Electrodeposited Sn-Zn alloy films were composed of a non-equilibrium phase, like an enforced solid solution, which was not observed in an equilibrium phase diagram of an Sn-Zn binary alloy system. By applying a thermal annealing process at 150 °C for 10 minutes, a pure Zn phase was precipitated from an electrodeposited Sn-based solid solution phase with excessively dissolved Zn atoms. During the soldering process, intermetallic phases such as Cu_3_Sn and Cu_5_Zn_8_ were formed at the interface between an Sn-Zn alloy and Cu substrate. Tensile strength and fracture elongation of solder-jointed Cu rods with Sn-8 at.%Zn alloy films reached ca. 40 MPa and 12%, respectively.

## 1. Introduction

As an environmentally adaptable technique, the development of a lead-free soldering process has become an important issue all over the world. In addition, the size of mobile electric devices such as smart phones and tablet PCs are becoming smaller and thinner year by year. Hence, development of a fine pitch joint soldering process is required for an industrial application. Electrochemical synthesis techniques of Sn based alloys from an aqueous solution have outdone all other techniques in the fabrication of fine pitch solder bumps due to its cost-effectiveness in mass production [1]. In a trend over time, several research works on the electrodeposition of lead-free solder materials such as Sn-Bi [1,2], Sn-Ag [3,4] and Sn-Cu [5,6,7] alloys have been conducted so far. Among their alloys, Sn-Zn alloys are attracting attention as a promising lead-free solder because of their cost performance, in having the closest melting point to Sn-Pb alloys in eutectic composition, and their excellent mechanical properties [8,9,10]. Furthermore, in the above Sn-based alloys, if alloying elements such as Cu^2+^ ions with a nobler standard electrode potential compared to Sn/Sn^2+^ (E^0^ = −0.138 V vs. NHE) are added to an electrolytic solution, Sn^2+^ ions will reduce Cu^2+^ ions to metallic Cu, and Cu^2+^ ions will oxidize Sn^2+^ ions to Sn^4+^ ions. These electrochemical reactions will result in the formation of a stannic acid precipitate in the electrolytic solution. The same phenomenon will also occur in Sn-Ag and Sn-Bi alloys. On the other hand, in Sn-Zn alloys, the standard electrode potential of Zn/Zn^2+^ (E^0^ = −0.763 V vs. NHE) is less nobler than that of Sn. Thus, any precipitations which are caused by a redox system of coexisting metal ions will not occur in an electrolytic solution containing Sn^2+^ and Zn^2+^ ions [11].

Based on an Sn-Zn binary alloy phase diagram, the eutectic temperature and composition are 198.5 °C and Sn-14.9 at.%Zn, respectively, while the melting points of pure Sn and pure Zn are 231.9 °C and 419.6 °C, respectively [12]. Motoyama et al. reported that the flow length of a liquid metal increased linearly with an increase in the superheat above the liquidus temperature [13]. Hence, it is estimated that the solder wettability of Sn-14.9 at.%Zn alloy will be better than that of pure Sn. However, in the electrodeposition of a binary alloy system, the standard electrode potentials are very influential on their co-deposition behavior. In Sn-Zn alloys, the difference between those standard electrode potentials is ca. 0.625 V. It is well known that the co-deposition of binary alloys will be quite difficult when the difference between each standard electrode potential is more than 0.3 V [1]. Until now, some researchers have reported the co-deposition behavior of Sn-Zn alloys from an aqueous solution containing some appropriate complexing agents. Andrew et al. revealed that Sn and Zn can be electrodeposited individually and as alloys from an electrolytic solution containing choline chloride and ethylene glycol or urea [14]. In addition, the electrodeposition of Sn-Zn alloys from an aqueous solution containing tartaric acid [15,16], citric acid [17,18,19], gluconic acid [20,21,22] and from an alkaline aqueous solution [23] has been investigated. While these research works revealed that Sn-Zn alloys can be electrodeposited from several types of electrolytic solutions, there are no reports on the solderability of electrodeposited Sn-Zn alloys.

In this study, Sn-Zn alloys were electrodeposited from an electrolytic solution containing citric acid as a complexing agent because the solution exhibited excellent stability at the neutral pH range, which is quite an advantage in applying to an industrial plant. To reveal the electrodeposition behavior of Sn-Zn alloys, alloy compositions, crystal structures and surface morphologies were investigated. Furthermore, the solderability of electrodeposited Sn-Zn alloys was also investigated by using Cu rods which were jointed with electrodeposited Sn-Zn alloy films.

## 2. Materials and Methods

The aqueous electrolytic solution was composed from SnSO_4_ (0.018 mol/L), ZnSO_4_·7H_2_O (0.182 mol/L), Na_3_C_6_H_5_O_7_·2H_2_O (0.25 mol/L), polyethylene glycol (PEG) (0.5 g/L) and sodium dodecyl sulfate (SDS) (0.05 g/L). The standard electrode potential value differs greatly between Sn and Zn. Hence, to make these metals co-deposit, suitable complexing agents and additives are required. In this study, trisodium citrate dehydrate (Na_3_C_6_H_5_O_7_) was used as a complexing agent. Further, polyethylene glycol (PEG) and sodium dodecyl sulfate (SDS) were used as additives to improve the surface smoothness. The electrolytic solution pH was adjusted to 5 by adding sulfuric acid because Sn^2+^ and Zn^2+^ can exist stably as their complex ions with citric acid in the solution pH range. A gold wire, a copper foil and a saturated Ag/AgCl electrode (DKK-TOA, Tokyo, Japan) were used for an anode, a cathode, and a reference electrode, respectively. The co-deposition behavior of Sn and Zn was evaluated by a cyclic voltammetry method to determine an optimum operating condition. During the evaluation, the cathode potential was swept from 0 to –2.0 V vs. Ag/AgCl by keeping the sweep rate at 20 mV/s. To make Sn-Zn alloy films, electrodeposition was performed galvanostatically at a cathode current density range between 0.5 and 15.0 mA/cm^2^. The electrolytic solution temperature during the electrodeposition was kept to 25 °C and the solution was stirred by a magnetic stirrer at a rotation speed of 200 rpm. The amount of charge during the electrodeposition was fixed to 30 C (coulomb).

**Figure 1:**
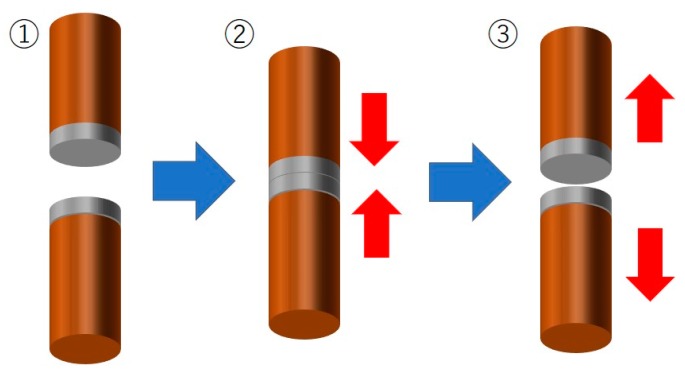
Experimental process to evaluate the solderability of electrodeposited Sn-Zn alloy films synthesized on Cu rods. (**1**): Electrodeposition of Sn-Zn alloy films on a cross-section of Cu rods. (**2**): Butt-joint of Cu rods by applying a constant pressure. (**3**): Tensile test to evaluate the solderability.

The surface morphology of electrodeposited Sn-Zn alloy films was evaluated by utilizing a scanning electron microscope (SEM, JEOL, Tokyo, Japan). The constituent phase of the alloy films was investigated by using an X-ray diffractometer (Rigaku, Tokyo, Japan). In addition, the chemical composition of the alloy films was analyzed by utilizing an energy dispersive X-ray analyzer, EDX (Shimadzu, Kyoto, Japan). Copper rods with a 10 mm diameter were used to evaluate the solderability of electrodeposited Sn-Zn alloy films. After mechanically polishing the cross-section of the copper rods, Sn-Zn alloy films were electrodeposited on the surface. Then, as shown in Figure 1, copper rods with alloy films were butt-jointed to each other by applying a constant pressure in a mold heated to 250 °C for 30 mins. After the bonding process, a tensile test was performed by utilizing the butt-jointed copper rods. During the tensile test, the crosshead speed was kept to 0.5 mm/min.

## 3. Results and Discussion

### 3.1. Electrodeposition Process of Sn-Zn Alloy Films

**Figure 2:**
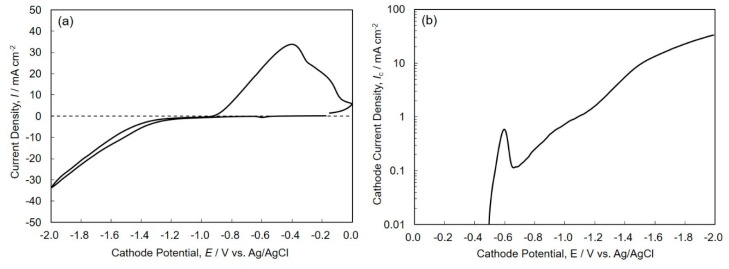
Cyclic voltammogram (**a**) and the Tafel plot (**b**) for electrodeposition of Sn-Zn alloys on a copper electrode.

Figure 2 shows a cyclic voltammogram (a) and the Tafel plot (b) for electrodeposition of Sn-Zn alloys on a copper electrode. According to the Nernst equation, the equilibrium potentials of Sn and Zn in an electrolytic solution without citric acid can be calculated as –0.391 V and –0.984 V vs. Ag/AgCl, respectively. As shown in Figure 2a, the remarkable rise of the cathodic and anodic current density are observed in the potential of ca. –1.2 V and –0.9 V, respectively. Considering the equilibrium potential of Zn, the cathodic and anodic current seem to be the reduction and dissolution current of Zn^2+^ ions. To investigate the detail in the electrochemical reduction behavior of Sn^2+^ ions, the Tafel plot was employed to enlarge the very small cathode current region. As shown in Figure 2b, the cathode current begins to increase at ca. –0.5 V, which is less noble than the equilibrium potential of Sn. It is well known that complex metal ions are reduced to a metallic state with an accompanying substantial overpotential [24]. Therefore, this increase in the cathode current seems to correspond to an electrochemical reduction current of complex Sn^2+^ ions with citric acid. With a sweeping of the cathode potential to a less noble direction, the cathode current density increases up at around 0.7 mA cm^-2^ and then decreases down to 0.1 mA cm^-2^. This phenomenon seems to be caused by the formation of complex Sn^2+^ ions with citric acid, which is more stable than the hydrated Sn^2+^ ions. Furthermore, with a sweeping of the cathode potential to ca. –1.2 V, which is less noble than the equilibrium potential of Zn, the cathode current density increases again. This increase in the cathode current seems to correspond to an electrochemical reduction current of complex Zn^2+^ ions with citric acid. Moreover, with sweeping of the cathode potential to the region less than –1.5 V, the slope of the polarization curve decreases because the diffusion of complex Zn^2+^ ions with citric acid reaches to the limit.

**Figure 3:**
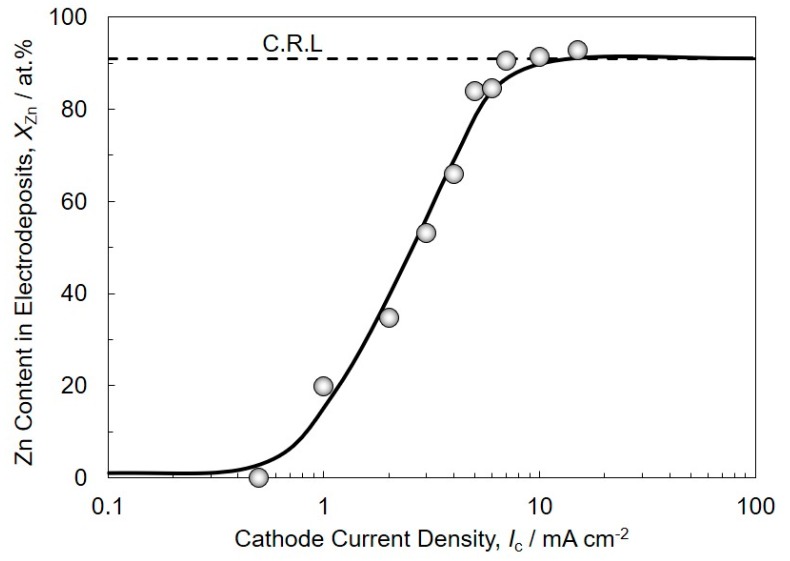
Effect of cathode current density on Zn content in electrodeposited Sn-Zn alloy films.

Figure 3 shows the effect of cathode current density on Zn content in the electrodeposited Sn-Zn alloy films. Zn content in the alloy films was investigated on the samples which were electrodeposited at the current density range from 0.5 to 15 mA/cm^2^. As the current density increased up to ca. 7 mA/cm^2^, Zn content in the alloy films increased and reached the composition reference line (C.R.L.) of 91 at.%, which is identical to the metallic ions ratio in the electrolytic solution. Over the current density range, at more than 7 mA/cm^2^, it is presumed that the diffusion of both Sn^2 +^ ions and Zn^2 +^ ions have reached the limit. This phenomenon corresponds to the typical normal co-deposition behavior in Brenner’s classification for the electrodeposition of binary alloys.

### 3.2. Surface Morphology and Constituent Phases of Electrodeposited Sn-Zn Alloy Films

**Figure 4:**
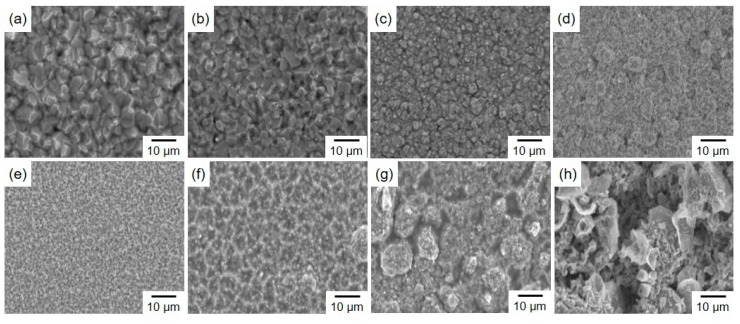
SEM image of the surface of the Sn-Zn alloy films (cathode current density; (**a**) 0.5 mA/cm^2^, (**b**) 1 mA/cm^2^, (**c**) 2 mA/cm^2^, (**d**) 3 mA/cm^2^, (**e**) 5 mA/cm^2^, (**f**) 7 mA/cm^2^, (**g**) 10 mA/cm^2^, (**h**) 15 mA/cm^2^).

Figure 4 shows the effect of current density on the surface morphology of electrodeposited Sn-Zn alloy films. Those SEM images (Figure 4a–h) were obtained from the Sn-Zn alloy films, which were electrodeposited at the current density of 0.5, 1, 2, 3, 5, 7, 10, and 15 mA/cm^2^, respectively. As shown in Figure 4a–e, in the low current density range from 0.5 to 5 mA/cm^2^, the average crystal grain size seems to decrease with an increase in the current density. While the nodule-like crystals, which are composed of small crystal grains, were observed exceptionally in Figure 4d. As shown in Figure 2b, the cathode potential shifts to a less noble direction with an increase in the current density over the range from 0.5 to 5 mA/cm^2^. It is well-known that the electrodeposited metals nucleation site density increases with an increase in the electrodeposition overpotential, which corresponds to the difference between the cathode potential and the equilibrium potential of metal, during the charge transfer process which is controlling the reduction rate of metallic ions [25]. Hence, the decrease in the average crystal grain size seems to be caused by the increase in the nucleation site density. On the other hand, as shown in Figure 4f–h, in the high current density range from 7 to 15 mA/cm^2^, the surface roughness seems to be enhanced with an increase in the current density. Furthermore, at the current density of 10 mA/cm^2^ or more, due to excessive hydrogen evolution, numerous pores were generated in the alloy films because the diffusion of both Sn^2 +^ ions and Zn^2 +^ ions have reached the limit as shown in Figure 3.

**Figure 5:**
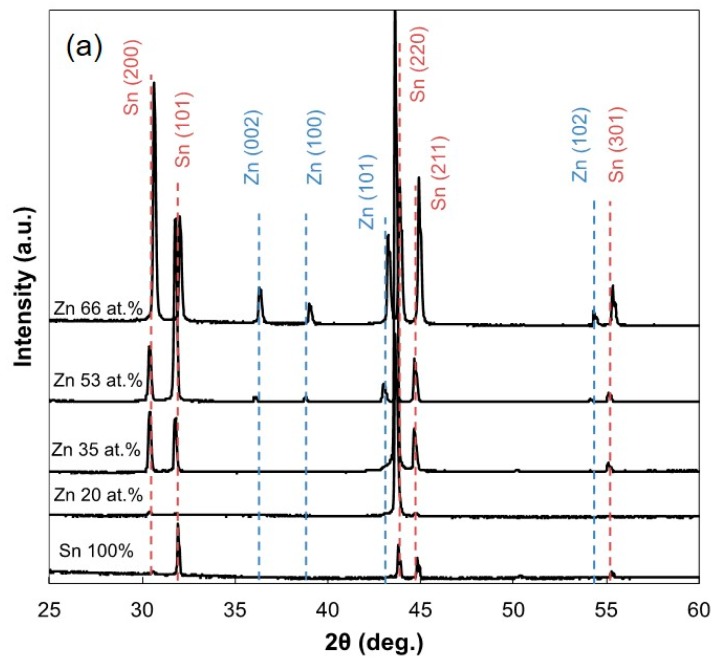

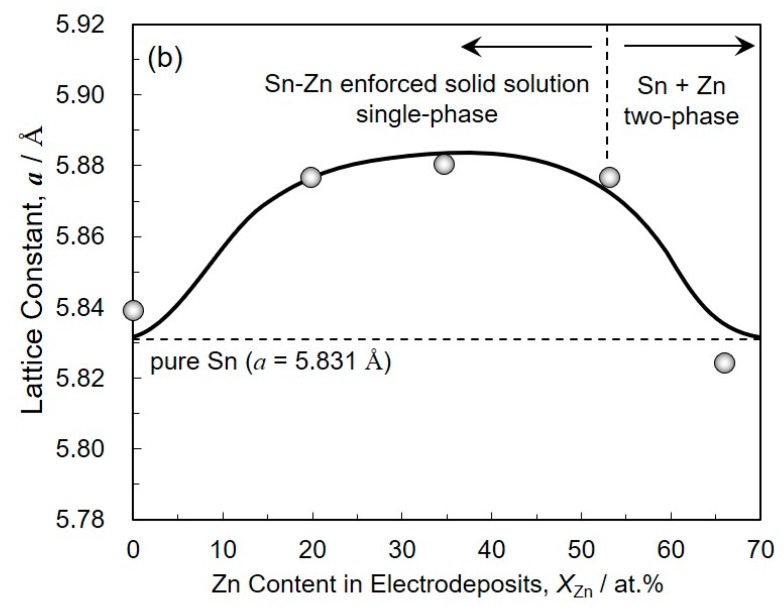
Effect of Zn content on X-ray diffraction profiles (**a**) and lattice constants (**b**) of electrodeposited Sn-Zn alloy films.

Figure 5 shows the effect of Zn content on the X-ray diffraction patterns (Figure 5a) and the lattice constants (Figure 5b) of electrodeposited Sn-Zn alloy films. As shown in Figure 5a, any peaks derived from Zn phases could not be observed in the samples with a Zn content of ca. 35 at.% or less. On the other hand, several peaks derived from Zn phases were observed in the samples with Zn content of ca. 53 at.% or more. However, the solubility limit of Zn atoms into the Sn-based solid-solution phase is only ca. 0.2 at.% at room temperature according to the Sn-Zn binary alloy phase diagram [12]. As shown in Figure 5b, with an increase in Zn content, the lattice constant also increased up to ca. 5.88 Å. Hence, in the electrodeposited Sn-Zn alloy films with a Zn content of 35 at.% or less, all Zn atoms seem to be dissolved in the Sn-based solid solution phase with its supersaturated atoms. On the other hand, there is also another reason which can explain the above results. It has been reported that electrodeposited metallic alloys are composed of extremely fine crystals [26]. Therefore, Zn atoms might be segregated at the grain boundaries of large Sn crystals and exist as very fine Zn crystals which are not able to be observed in X-ray diffraction patterns.

**Figure 6:**
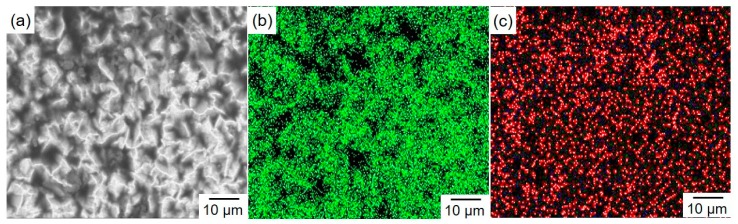
FE-SEM image of an electrodeposited Sn-15 at.%Zn alloy film (**a**). EDX elemental mapping images of Sn and Zn are also shown in (**b**) and (**c**), respectively.

To confirm the above estimation concerning the solubility of Zn atoms, elemental mapping analysis was performed by using an FE-SEM/EDX analyzer. Figure 6a shows the FE-SEM image of an electrodeposited Sn-15 at.% Zn alloy film. EDX elemental mapping images of Sn and Zn are also shown in Figure 6b,c, respectively. According to Figure 6b,c, it is revealed that Zn atoms exist over a wide range and in the same sites as Sn atoms. Hence, Zn atoms seem to be dissolved in the Sn-based solid solution phase with its supersaturated atoms.

**Figure 7:**
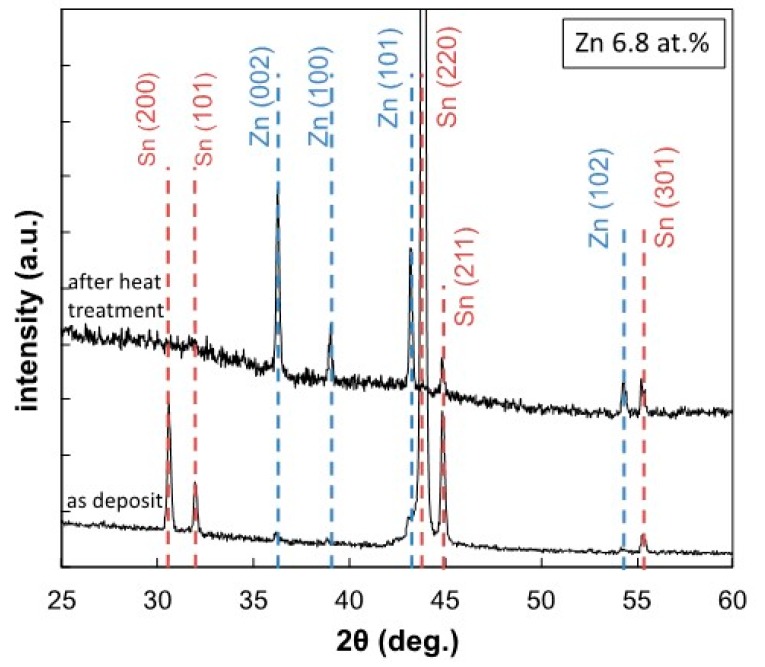
Effect of thermal annealing on the XRD profiles of an electrodeposited Sn-6.8 at.%Zn alloy film.

It is well known that the thermodynamic non-equilibrium phases such as supersaturated solid solution or amorphous alloys will precipitate the excessively dissolved atoms and transform to stable phases when they are annealed at a temperature below the melting point [27]. Therefore, to investigate the phase transformation performance of the enforced solid solution phase, a thermal annealing process was applied to an electrodeposited Sn-Zn alloy film, which was exfoliated from the cathode substrate. Figure 7 shows the effect of thermal annealing at 150 °C for 10 minutes on the XRD profiles of an electrodeposited Sn-6.8 at.%Zn alloy film. Before the thermal annealing process, the large diffraction peaks, which were derived from an Sn-based solid solution phase, were clearly observed in addition to the very small peaks that would correspond to a pure Zn phase. After the thermal annealing process, several diffraction peaks, which were derived from a pure Zn phase, were also observed in addition to the Sn-based solid solution phase. This result seems to be caused by the precipitation of a pure Zn phase from the Sn-based solid solution phase with excessively dissolved Zn atoms by the thermal annealing process.

### 3.3. Soldering Performance of Electrodeposited Sn-Zn Alloy Films

**Figure 8:**
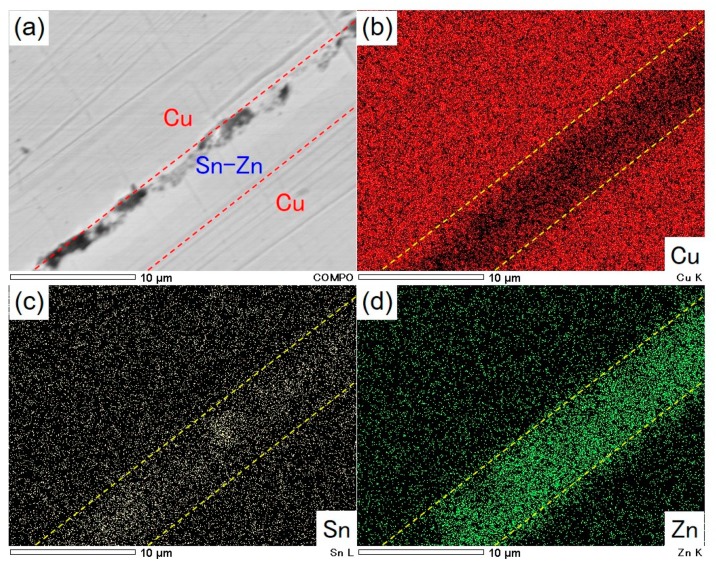
Cross-sectional SEM image of copper rods that were solder-jointed with an electrodeposited Sn-39 at.%Zn alloy film (**a**) EDX elemental mapping images of Cu, Sn and Zn are also shown in (**b**), (**c**) and (**d**), respectively.

Figure 8 shows a cross-sectional SEM image of copper rods that were solder-jointed with an electrodeposited Sn-39 at.%Zn alloy film (a), EDX elemental mapping images of Cu, Sn and Zn are also shown in Figure 8b–d, respectively. As shown in this figure, each copper rod seems to be tightly jointed via the Sn-Zn alloy layer and the layer thickness was ca. 5.8 µm. By using the solder-jointed copper rods, the following tensile test was performed to evaluate the mechanical properties.

**Figure 9:**
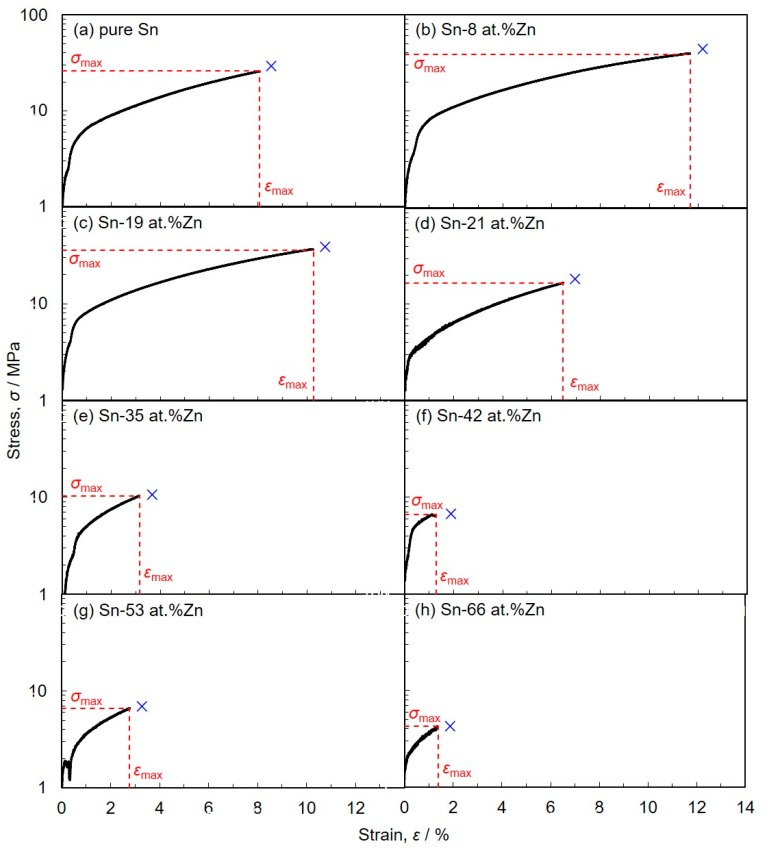
Stress–strain curves obtained from butt-jointed copper rods which were soldered with electrodeposited Sn-Zn alloy films. (Zn content: (**a**) 0 at.%, (**b**) 8 at.%, (**c**) 19 at.%, (**d**) 21 at.%, (**e**) 35 at.%, (**f**) 42 at.%, (**g**) 53 at.%, (**h**) 66 at.%)

Figure 9 shows the stress–strain curves obtained from butt-jointed copper rods which were soldered with electrodeposited Sn-Zn alloy films. As shown in Figure 9, the stress–strain performance of the solder-jointed copper rods was strongly affected by the composition of Sn-Zn alloy films. In Zn content less than 20 at.%, the tensile strength, *σ*_max_ and fracture elongation, *ε*_max_ were larger than those of copper rods which were soldered with an electrodeposited pure Sn film. Especially, in Sn-8 at.%Zn alloy, *σ*_max_ and *ε*_max_ reached ca. 40 MPa and 12%, respectively. While, in Zn content more than 20 at.%, *σ*_max_ and *ε*_max_ were smaller than those of solder-jointed copper rods with pure Sn film. Zhang et al. reported that the mechanical properties of copper joints which were reflow-soldered with Sn-3 wt.%Ag-0.5wt.%Cu commercial paste [28]. In the report, they found that the tensile strength was ca. 50 MPa. Li et al. also reported that the mechanical performance of copper joints which were ultrasonically soldered with Sn-3 wt.%Ag-0.5 wt.%Cu alloy bulk foils [29]. In the article, they revealed that the shear strength was ca. 40 MPa. Hence, it was revealed that the soldering performance of electrodeposited Sn-Zn alloy films has been approaching to the practical level in Sn-Ag-Cu alloy solders.

**Figure 10:**
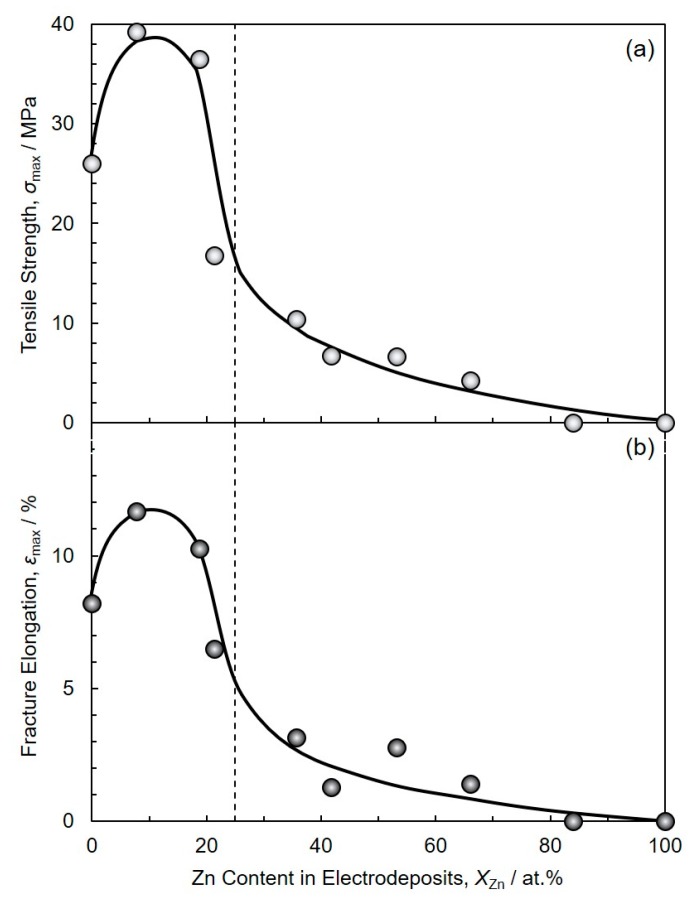
Effect of alloy composition on the tensile strength (**a**) and fracture elongation (**b**) of a pair of Cu rods which are jointed with electrodeposited Sn-Zn alloy films in the cross-section.

Figure 10 shows the effect of Zn content in electrodeposits, *X*_Zn_ on the tensile strength, *σ*_max_ (a) and fracture elongation, *ε*_max_ (b) of a pair of Cu rods which are jointed with electrodeposited Sn-Zn alloy films in the cross-section. Cu rods were not joined by the alloy films with *X*_Zn_ more than ca. 80 at.%. Hence, the tensile tests for those samples were not able to be performed. As shown in Figure 10a, in the range of Zn content less than 20 at.% (*X*_Zn_ < 20 at.%), *σ*_max_ and *ε*_max_ were greater than those of copper rods with pure Sn film (*X*_Zn_ = 0 at.%). While, *σ*_max_ and *ε*_max_ decreased with increasing *X*_Zn_ over the range from ca. 20 at.% to ca. 80 at.%. Based on the Sn-Zn binary alloy phase diagram at 250 °C, the liquid phase is stable at *X*_Zn_ range from 0 at.% to ca. 25 at.% [12]. While, in *X*_Zn_ range more than ca. 25 at.%, the volume ratio of liquid phase, *R*^L^ can be described by the following equation.

(1) $$R^{L}=\frac{X_{}^{S}-X_{Zn}}{X_{}^{S}-X_{}^{L}}=\frac{100-X_{Zn}}{100-25}$$

Here, *X*^S^ and *X*^L^ are the equilibrium Zn contents of solid phase and liquid phase at 250 °C, respectively. *X*^S^ and *X*^L^ are determined to ca. 100 at.% and ca. 25 at.% from Sn-Zn binary alloy phase diagram, respectively. While, *X*_Zn_ is Zn content of electrodeposited Sn-Zn alloy films. According to the above equation, *R*^L^ decreases with an increase in *X*_Zn_. Hence, according to Figure 10, *σ*_max_ and *ε*_max_ seem to depend on *R*^L^ at 250 °C. On the other hand, as shown in Figure 4, the average crystal grain size decreased with an increase in *X*_Zn_ up to ca. 80 at.%. However, during the soldering process, crystal grain growth and solid–liquid phase transformation will proceed drastically. Therefore, we could not reveal the effect of crystal grain size on the tensile strength.

**Figure 11:**
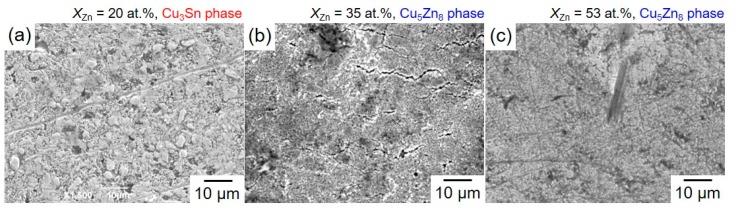
SEM images of the fracture surface of the solder-jointed copper rods with Sn-Zn alloy films (Zn content; (**a**) 20 at.%, (**b**) 35 at.%, (**c**) 53 at.%).

Figure 11 shows SEM images of the fracture surface, which were obtained after the tensile test by utilizing the solder-jointed Cu rods with Sn-Zn alloy films (Zn content; (a) 20 at.%, (b) 35 at.%, (c) 53 at.%). As shown in Figure 11a, in the alloy film with Zn content of 20 at.%, the surface was very rough due to the typical ductile fracture. While, in the alloy film with Zn content of 53 at.%, the surface was very flat due to the typical brittle fracture as shown in Figure 11c.

**Figure 12:**
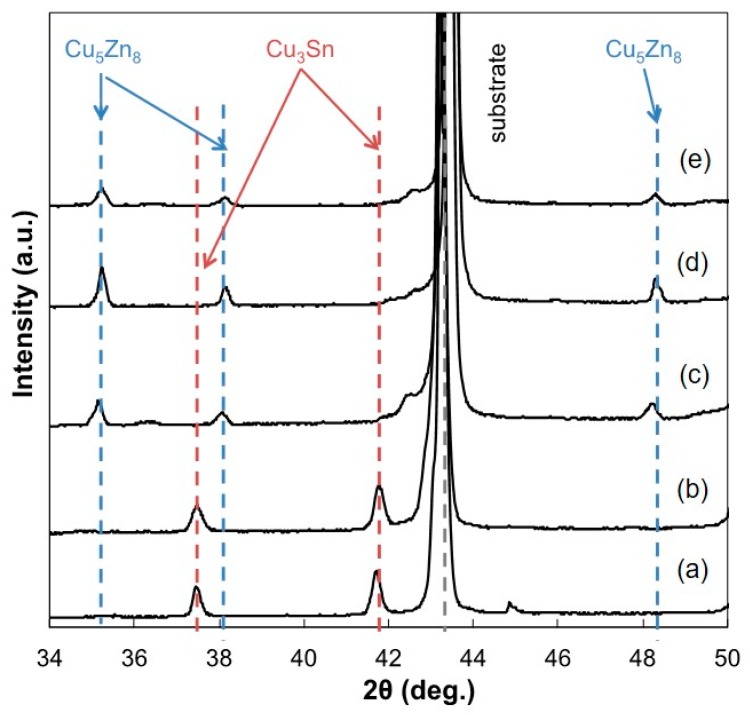
X-ray diffraction patterns which were obtained from the fracture surfaces of the jointed Cu rods after the tensile test. (Zn content: (**a**) 0 at.%, (**b**) 20 at.%, (**c**) 35 at.%, (**d**) 53 at.%, (**e**) 66 at.%).

Figure 12 shows X-ray diffraction patterns, which were obtained from the fracture surfaces of the jointed Cu rods after the tensile test as shown in Figure 9. From these diffraction patterns, it was revealed that the electrodeposited Sn and Zn had formed some intermetallic compounds with the Cu substrate at 250 °C. For example, the diffraction peaks, which were derived from the Cu_3_Sn intermetallic compound phase, were observed from the fracture surfaces that were coated by electrodeposited Sn-Zn alloy films with zinc content, *X*_Zn_ no more than 20 at.%. Based on the Cu-Sn binary alloy phase diagram, orthorhombic *ε* phase (Cu_3_Sn) is stable at Sn content range from 24.5 to 25.9 at.% [30]. Chia et al. also reported that Cu_3_Sn phase was observed in the interface between an electrodeposited Sn film and Cu substrate, which were reflowed at 300 °C for 60 minutes [31]. While the diffraction peaks, which were derived from Cu_5_Zn_8_ intermetallic compound phase, were also observed from the samples with *X*_Zn_ no less than 35 at.%. Luan et al. reported that Cu_5_Zn_8_ intermetallic compound phase was observed from the interfaces between Cu substrates and bulk filler Sn-Zn alloys with *X*_Zn_ more than 1 wt.% [32]. They found that the joints with a Cu_5_Zn_8_ intermetallic compound phase exhibit a higher strength than those with a Cu_6_Sn_5_ intermetallic compound phase. While, in the present study, Cu_5_Zn_8_ intermetallic compound phases were not observed from the samples with *X*_Zn_ at no more than 20 at.%.

## 4. Conclusions

The electrodeposition process of Sn-Zn alloys from an aqueous solution containing citric acid complexes corresponds to the typical normal co-deposition behavior in Brenner’s classification. In the electrodeposited Sn-Zn alloy films with a Zn content of no more than 35 at.%, Zn atoms were dissolved in Sn-based solid solution phase with its supersaturated atoms. By applying a thermal annealing process at 150 °C for 10 min, a pure Zn phase was precipitated from an electrodeposited Sn-based solid solution phase with excessively dissolved Zn atoms. In the range of *X*_Zn_ less than 20 at.%, *σ*_max_ and *ε*_max_ of the solder-jointed Cu rods were larger than those of Cu rods with pure Sn film. Especially, in Sn-8 at.%Zn alloy, *σ*_max_ and *ε*_max_ reached ca. 40 MPa and 12%, respectively. While, *σ*_max_ and *ε*_max_ decreased with increasing *X*_Zn_ over the range from ca. 20 at.% to ca. 80 at.%. Based on a Sn-Zn binary alloy phase diagram at 250 °C, it was suggested that the tensile strength decreases with a decreasing of the volume ratio of liquid phase in *X*_Zn_ more than ca. 25 at.%. After the tensile test of solder jointed Cu rods, a Cu_3_Sn intermetallic compound phase was observed from the samples with *X*_Zn_ at no more than 20 at.%. These results in the present study will open up a new possibility to develop a Pb-free soldering system in microelectronics.
